# Community-setting pneumonia-associated hospitalizations by level of urbanization—New York City versus other areas of New York State, 2010–2014

**DOI:** 10.1371/journal.pone.0244367

**Published:** 2020-12-23

**Authors:** Melody Wu, Katherine Whittemore, Chaorui C. Huang, Rachel E. Corrado, Gretchen M. Culp, Sungwoo Lim, Neil W. Schluger, Demetre C. Daskalakis, David E. Lucero, Neil M. Vora

**Affiliations:** 1 Department of Epidemiology, Mailman School of Public Health, Columbia University, New York, New York, United States of America; 2 Division of Disease Control, New York City Department of Health and Mental Hygiene, Queens, New York, United States of America; 3 Division of Epidemiology, New York City Department of Health and Mental Hygiene, Queens, New York, United States of America; 4 Division of Pulmonary, Allergy and Critical Care Medicine, Columbia University Medical Center, New York, New York, United States of America; 5 Departments of Medicine, Epidemiology and Environmental Health Science, Columbia University Vagelos College of Physicians and Surgeons and Mailman School of Public Health, New York, New York, United States of America; 6 Career Epidemiology Field Officer Program, Center for Preparedness and Response, Centers for Disease Control and Prevention, Atlanta, Georgia, United States of America; BronxCare Health System, Affiliated with Icahn School of Medicine at Mount Sinai, UNITED STATES

## Abstract

**Background:**

New York City (NYC) reported a higher pneumonia and influenza death rate than the rest of New York State during 2010–2014. Most NYC pneumonia and influenza deaths are attributed to pneumonia caused by infection acquired in the community, and these deaths typically occur in hospitals.

**Methods:**

We identified hospitalizations of New York State residents aged ≥20 years discharged from New York State hospitals during 2010–2014 with a principal diagnosis of community-setting pneumonia or a secondary diagnosis of community-setting pneumonia if the principal diagnosis was respiratory failure or sepsis. We examined mean annual age-adjusted community-setting pneumonia-associated hospitalization (CSPAH) rates and proportion of CSPAH with in-hospital death, overall and by sociodemographic group, and produced a multivariable negative binomial model to assess hospitalization rate ratios.

**Results:**

Compared with non-NYC urban, suburban, and rural areas of New York State, NYC had the highest mean annual age-adjusted CSPAH rate at 475.3 per 100,000 population and the highest percentage of CSPAH with in-hospital death at 13.7%. NYC also had the highest proportion of CSPAH patients residing in higher-poverty-level areas. Adjusting for age, sex, and area-based poverty, NYC residents experienced 1.3 (95% confidence interval [CI], 1.2–1.4), non-NYC urban residents 1.4 (95% CI, 1.3–1.6), and suburban residents 1.2 (95% CI, 1.1–1.3) times the rate of CSPAH than rural residents.

**Conclusions:**

In New York State, NYC as well as other urban areas and suburban areas had higher rates of CSPAH than rural areas. Further research is needed into drivers of CSPAH deaths, which may be associated with poverty.

## Introduction

While pneumonia and influenza (P&I) together ranked as the third-leading cause of death in New York City (NYC) during 2010–2014, they were the sixth- to seventh-leading cause of death in New York State (NYS) excluding NYC [1, 2]. In 2014, the age-adjusted P&I death rate was 24.9 per 100,000 population in NYC versus 16.0 in the rest of NYS and 15.1 in the nation [1–3]. Most P&I deaths in NYC are attributed to pneumonia, and pneumonia deaths typically occur in hospitals [4–6].

Reasons why the P&I mortality rate is higher in NYC than elsewhere in the state are unknown. There may be differences in clinical care, though we are unaware of data indicating that this is occurring [5]. Worse ambient pollution is associated with increased pneumonia deaths, and the NYC metro area is one of the most polluted for ozone in the United States (U.S.) [7, 8]. In addition, it is possible that NYC hospitals admit a greater proportion of patients with more severe pneumonia. Although hospital-acquired or ventilator-associated pneumonia are associated with increased mortality and are more frequently caused by antimicrobial-resistant bacteria than community-acquired pneumonia (CAP), they are unlikely to be driving the higher death rate in NYC because the majority of pneumonia-associated hospitalizations and deaths from such hospitalizations are due to community-setting pneumonia, i.e., either CAP or healthcare-associated pneumonia (HCAP) [4, 9, 10]. HCAP refers to pneumonia that is associated with exposure to healthcare settings and typically has onset when a person is in the community rather than a hospital. Finally, findings of higher mortality in NYC may be artifactual; for example, NYC physicians may be more likely to list pneumonia as the underlying cause of death on the death certificate than non-NYC physicians, but we are unaware of data that indicate such differences in coding practice.

Limited data exist about the burden of community-setting pneumonia-associated hospitalizations (CSPAH) across NYS. It is important to assess hospitalization rates more locally than the state level because health disparities have been identified within states [11]. Furthermore, geographic areas lying on different points of the rural-urban continuum have different population densities, which may influence the effective contact rate of pneumonia pathogens [12]. These areas may also have different patient population mixes, level of healthcare access, and quality of pneumonia treatment [13–17]. Thus, the objective of this study was to compare CSPAH across NYS by urbanization level (NYC, non-NYC urban areas, suburban areas, and rural areas).

## Materials and methods

### Study approvals

The study was determined to be exempt research by Columbia University, the NYC Department of Health and Mental Hygiene, and the Centers for Disease Control and Prevention.

### Data source and study population

We analyzed discharge data for NYS residents aged ≥20 years who were hospitalized in NYS acute care facilities during January 1, 2010–December 31, 2014 using the Statewide Planning and Research Cooperative System (SPARCS). SPARCS is a comprehensive all-payer reporting system that collects patient-level administrative data on every inpatient stay in hospitals regulated by New York Public Health Law Article 28 [18, 19]. Records with unknown patient sex, records where the patient was discharged or transferred to inpatient care, and records that closely followed a previously captured hospitalization were excluded from the study.

### Definitions

We defined CSPAH as hospitalizations with a principal diagnosis of pneumonia (International Classification of Diseases, Ninth Revision, Clinical Modification [ICD-9-CM] codes 480–486, 487.0, 488.01, 488.11, 488.81) or a secondary diagnosis of pneumonia if the principal diagnosis was respiratory failure (ICD-9-CM codes 518.81, 518.82, 518.84, 799.1) or sepsis (ICD-9-CM codes 038, 785.52, 995.91, 995.92) [4, 20]. Community-setting pneumonia also had to meet the Corrado et al. study definition for CAP or HCAP, both of which develop outside of the hospital; these definitions were adapted from Infectious Disease Society of America and American Thoracic Society guidelines [4]. While the designation of some pneumonias as HCAP has fallen out of favor, we decided to retain that designation in this analysis for consistency with previously published methodology [4, 9, 21].

### Data analysis

The unit of analysis was a hospitalization. We compared CSPAH by urbanization level across NYS using Rural-Urban Continuum Codes (RUCC) based on county of patient residence in NYC (RUCC 1), non-NYC urban areas (RUCC 1–3 other than NYC), suburban areas (RUCC 4–5), or rural areas (RUCC 6–8). For 3,754 CSPAH records missing information needed to determine urbanization level, we used the rUSPS package in R to verify and format addresses and then geocoded with the rGBAT package. Approximately 30% of the 3,754 records belonged to non-NYC homeless patients and could not be geocoded; these records were excluded and represented <0.5% of the total eligible CSPAH.

We examined data on CSPAH and death during CSPAH by sex, age, race/ethnicity, area-based poverty level, setting of pneumonia acquisition (i.e., CAP or HCAP), length of stay, primary source of payment, van Walraven comorbidity index score, listing of HIV/AIDS as a secondary diagnosis (ICD-9-CM codes 042, 079.53, V08, 795.71), and listing of chronic obstructive pulmonary disease (COPD) as a secondary diagnosis (ICD-9-CM codes 491.0, 491.1, 491.20, 491.21, 491.8, 491.9, 492.0, 492.8, 494, 494.0, 494.1, 496). We included HIV/AIDS and COPD because they are important risk factors for pneumonia. The van Walraven comorbidity index accounts for certain co-factors of morbidity and mortality such as heart failure [22, 23]. Area-based poverty level was determined by ZIP Code of residence and categorized as: low (<10% of residents live below federal poverty level [FPL]), medium (10%–19% FPL), high (20%–29% FPL), and very high (≥30% FPL). Population estimates for area-based poverty level were determined by ZIP Code Tabulation Area.

Population estimates for the 62 NYS counties were extracted from the U.S. Census Bureau’s American Community Survey using five-year estimates and assuming the same population for each year from 2010–2014 [24]. We generated age-adjusted CSPAH rates using the direct method based on the 2000 U.S. Standard Population [25]. Rate ratios (RRs) and 95% confidence intervals (CIs) were calculated using negative binomial regression models, with p <0.05 being set as statistically significant. McFadden’s pseudo R^2^ was calculated to assess final model fit for the multivariable negative binomial regression model. Records with missing area-based poverty level were excluded from the analyses.

All analyses were conducted with RStudio Version 1.0.143 (RStudio, Inc., Boston, MA).

## Results

Of 10,532,246 total hospitalizations in NYS during 2010–2014, we identified 379,116 (3.6%) CSPAH. Of the 379,116 CSPAH, 40.3% (n = 152,643) were in NYC residents, 50.4% (n = 190,895) in non-NYC urban residents, 6.0% (n = 22,624) in suburban residents, and 3.4% (n = 12,954) in rural residents. (Table 1) Non-NYC urban areas and NYC had the highest percentages of HCAP at 31.5% and 31.4%, respectively, and rural areas the lowest at 27.4%.

**Table 1:** Characteristics of community-setting pneumonia-associated hospitalizations and associated in-hospital deaths in New York City, non-New York City urban areas, suburban areas, and rural areas—New York State, 2010–2014^a^.

	NYC		Non-NYC urban areas		Suburban areas		Rural areas	
Characteristic	No. of CSPAH (%)	No. of in-hospital deaths during CSPAH (%)	No. of CSPAH (%)	No. of in-hospital deaths during CSPAH (%)	No. of CSPAH (%)	No. of in-hospital deaths during CSPAH (%)	No. of CSPAH (%)	No. of in-hospital deaths during CSPAH (%)
Overall	152,643 (--)	20,842 (--)	190,895 (--)	20,371 (--)	22,624 (--)	1,960 (--)	12,954 (--)	1,147 (--)
Sex								
Female	78,643 (51.5)	10,611 (50.9)	98,572 (51.6)	10,264 (50.4)	11,239 (49.7)	973 (49.6)	6,312 (48.7)	559 (48.7)
Male	74,000 (48.5)	10,231 (49.1)	92,323 (48.4)	10,107 (49.6)	11,385 (50.3)	987 (50.4)	6,642 (51.3)	588 (51.3)
Age (years)								
20–44	15,148 (9.9)	583 (2.8)	13,588 (7.1)	390 (1.9)	1,517 (6.7)	41 (2.1)	786 (6.1)	23 (2.0)
45–64	41,660 (27.3)	3,692 (17.7)	46,019 (24.1)	3,213 (15.8)	5,598 (24.7)	324 (16.5)	3,164 (24.4)	179 (15.6)
65–79	44,060 (28.9)	6,233 (29.9)	58,760 (30.8)	6,522 (32.0)	7,764 (34.3)	698 (35.6)	4,359 (33.6)	391 (34.1)
≥80	51,775 (33.9)	10,334 (49.6)	72,528 (38.0)	10,246 (50.3)	7,745 (34.2)	897 (45.8)	4,645 (35.9)	554 (48.3)
Race/ethnicity								
NH White	54,199 (35.5)	8,857 (42.5)	149,349 (78.2)	16,362 (80.3)	18,594 (82.2)	1,659 (84.6)	11,175 (86.3)	991 (86.4)
NH Black	39,304 (25.7)	5,032 (24.1)	16,790 (8.8)	1,562 (7.7)	353 (1.6)	28 (1.4)	156 (1.2)	14 (1.2)
Hispanic	28,162 (18.4)	2,966 (14.2)	9,552 (5.0)	851 (4.2)	1,925 (8.5)	171 (8.7)	310 (2.4)	35 (3.1)
NH AI/AN	410 (0.3)	37 (0.2)	320 (0.2)	30 (0.1)	114 (0.5)	8 (0.4)	60 (0.5)	5 (0.4)
NH A/PI	8,549 (5.6)	1,330 (6.4)	1,579 (0.8)	182 (0.9)	20 (0.1)	0 (0.0)	15 (0.1)	1 (0.1)
NH other	14,702 (9.6)	1,722 (8.3)	5,054 (2.6)	552 (2.7)	180 (0.8)	24 (1.2)	195 (1.5)	33 (2.9)
Unknown	7,317 (4.8)	898 (4.3)	8,251 (4.3)	832 (4.1)	1,438 (6.4)	70 (3.6)	1,043 (8.1)	68 (5.9)
Area-based poverty level								
Low (<10% FPL)	23,528 (15.4)	3,455 (16.6)	106,366 (55.7)	11,809 (58.0)	3,545 (15.7)	325 (16.6)	2,324 (17.9)	194 (16.9)
Medium (10%–19% FPL)	52,845 (34.6)	7,590 (36.4)	49,363 (25.9)	5,237 (25.7)	13,800 (61.0)	1,210 (61.7)	8,734 (67.4)	760 (66.3)
High (20%–29% FPL)	41,795 (27.4)	5,785 (27.8)	21,336 (11.2)	2,061 (10.1)	4,608 (20.4)	371 (18.9)	1,592 (12.3)	165 (14.4)
Very high (≥30% FPL)	34,091 (22.3)	3,965 (19.0)	12,936 (6.8)	1,170 (5.7)	591 (2.6)	47 (2.4)	144 (1.1)	11 (1.0)
Unknown	384 (0.3)	47 (0.2)	894 (0.5)	94 (0.5)	80 (0.4)	7 (0.4)	160 (1.2)	17 (1.5)
Pneumonia setting of acquisition^b^								
CAP	104,746 (68.6)	10,795 (51.8)	130,790 (68.5)	10,402 (51.1)	16,147 (71.4)	1,075 (54.8)	9,401 (72.6)	700 (61.0)
HCAP	47,897 (31.4)	10,047 (48.2)	60,105 (31.5)	9,969 (48.9)	6,477 (28.6)	885 (45.2)	3,553 (27.4)	447 (39.0)
Length of stay (days)								
0–6	82,194 (53.8)	9,523 (45.7)	113,196 (59.3)	10,414 (51.1)	14,860 (65.7)	1,105 (56.4)	9,123 (70.4)	692 (60.3)
7–20	55,806 (36.6)	7,536 (36.2)	66,503 (34.8)	7,394 (36.3)	6,957 (30.8)	684 (34.9)	3,452 (26.6)	370 (32.3)
≥21	14,643 (9.6)	3,783 (18.2)	11,196 (5.9)	2,563 (12.6)	807 (3.6)	171 (8.7)	379 (2.9)	85 (7.4)
Primary source of payment								
Medicaid	18,843 (12.3)	1,645 (7.9)	10,749 (5.6)	680 (3.3)	1,296 (5.7)	69 (3.5)	661 (5.1)	22 (1.9)
Medicare	91,631 (60.0)	15,339 (73.6)	127,446 (66.8)	15,452 (75.9)	14,590 (64.5)	1,402 (71.5)	8,874 (68.5)	878 (76.5)
Private	38,255 (25.1)	3,639 (17.5)	43,263 (22.7)	3,446 (16.9)	5,212 (23)	355 (18.1)	2,640 (20.4)	166 (14.5)
Self-pay	3,079 (2.0)	169 (0.8)	7,560 (4.0)	604 (3.0)	1,118 (4.9)	101 (5.2)	480 (3.7)	42 (3.7)
Other	398 (0.3)	16 (0.1)	1,749 (0.9)	182 (0.9)	395 (1.7)	32 (1.6)	292 (2.3)	38 (3.3)
Unknown	437 (0.3)	34 (0.2)	128 (0.1)	7 (0.0)	13 (0.1)	1 (0.1)	7 (0.1)	1 (0.1)
van Walraven comorbidity index score^c^							
(16)–(1)^d^	8,832 (5.8)	146 (0.7)	9,084 (4.8)	150 (0.7)	1,230 (5.4)	17 (0.9)	684 (5.3)	16 (1.4)
0–15	102,332 (67.0)	9,869 (47.4)	127,825 (67.0)	9,235 (45.3)	15,413 (68.1)	903 (46.1)	9,408 (72.6)	623 (54.3)
16–31	38,972 (25.5)	9,749 (46.8)	51,114 (26.8)	9,922 (48.7)	5,707 (25.2)	946 (48.3)	2,763 (21.3)	473 (41.2)
32–47	2,467 (1.6)	1,054 (5.1)	2,840 (1.5)	1,051 (5.2)	271 (1.2)	92 (4.7)	96 (0.7)	33 (2.9)
48–63	40 (0.0)	24 (0.1)	32 (0.0)	13 (0.1)	3 (0.0)	2 (0.1)	3 (0.0)	2 (0.2)
HIV/AIDS								
Yes	7,734 (5.1)	529 (2.5)	1,773 (0.9)	96 (0.5)	60 (0.3)	4 (0.2)	50 (0.4)	3 (0.3)
No	144,909 (94.9)	20,313 (97.5)	189,122 (99.1)	20,275 (99.5)	22,564 (99.7)	1,956 (99.8)	12,904 (99.6)	1,144 (99.7)
COPD								
Yes	31,557 (20.7)	4,379 (21.0)	68,304 (35.8)	7,228 (35.5)	10,201 (45.1)	867 (44.2)	5,375 (41.5)	467 (40.7)
No	121,086 (79.3)	16,463 (79.0)	122,591 (64.2)	13,143 (64.5)	12,423 (54.9)	1,093 (55.8)	7,579 (58.5)	680 (59.3)

Abbreviations: AI/AN, American Indian/Alaskan Native; A/PI, Asian/Pacific Islander; CAP, community-acquired pneumonia; COPD, chronic obstructive pulmonary disease; CSPAH, community-setting pneumonia-associated hospitalizations; FPL, federal poverty level; HCAP, health-care-associated pneumonia; NH, non-Hispanic; NYC, New York City.

^a^Urbanization groups across New York State were classified using Rural-Urban Continuum Codes (RUCC) based on county of patient residence in NYC (RUCC 1), non-NYC urban areas (RUCC 1–3 other than NYC), suburban areas (RUCC 4–5), or rural areas (RUCC 6–8).

^b^CAP and HCAP both develop outside the hospital setting and compose CSPAH.

^c^The van Walraven comorbidity index score is a single number based on the strength of association between comorbidity groups and in-hospital death.

^d^These parentheses indicate negative values.

Increasingly older age groups represented increasingly larger proportions of CSPAH everywhere except for suburban areas, where those aged 65–79 years and aged ≥80 years each composed 34% of CSPAH. (Table 1) Non-Hispanic White patients represented the largest proportion of CSPAH among all racial/ethnic groups in each urbanization group, whereas non-Hispanic American Indian/Alaskan Native and non-Hispanic Asian/Pacific Islander patients generally represented the smallest proportions.

The majority of CSPAH in suburban and rural areas occurred among patients residing in medium-poverty-level areas, whereas the majority of CSPAH in non-NYC urban areas occurred among patients from low-poverty-level areas. In NYC, CSPAH were more evenly distributed among residents of medium- to very-high-poverty-level areas. NYC had the highest percentage of CSPAH occurring among residents of very-high-poverty-level areas (22.3%; non-NYC urban areas: 6.8%; suburban areas: 2.6%; and rural areas: 1.1%).

The distribution of van Walraven comorbidity index scores were similar across the four urbanization groups. NYC had the highest percentage of CSPAH lasting ≥21 days (9.6%) and non-NYC urban areas the second highest (5.9%). NYC also had the highest percentage of CSPAH with a secondary diagnosis of HIV/AIDS (5.1%) in NYS, compared with suburban areas at 0.3%, but the lowest percentage with a secondary diagnosis of COPD (20.7%; compared with suburban areas at 45.1%).

### In-hospital death

Death occurred during 11.7% of CSPAH across NYS (n = 44,320). Among NYC residents, 13.7% of CSPAH resulted in death, as did 10.7% among non-NYC urban residents, 8.7% among suburban residents, and 8.9% among rural residents. In every urbanization group, the percentage of CSPAH with in-hospital death that was associated with HCAP versus CAP was higher than the percentage of overall CSPAH that was associated with HCAP versus CAP (e.g., in NYC, 48.2% of CSPAH with in-hospital death were HCAP-related while 31.4% of CSPAH were HCAP-related) (Table 1).

Sociodemographic patterns in CSPAH deaths follow the hospitalization patterns described above except that increasingly older age groups represented increasingly larger proportions of in-hospital death in every urbanization group. (Table 1) NYC had a higher percentage of in-hospital death occurring among residents of high- and very-high-poverty-level areas with CSPAH (27.8% and 19.0%, respectively) compared with the rest of the state. Non-NYC urban areas had the lowest percentage of in-hospital death occurring among residents of high-poverty-level areas (10.1%), and rural areas had the lowest percentage of in-hospital death occurring among residents of very-high-poverty-level areas (1.0%).

### Mean annual age-adjusted CSPAH rates

Mean annual age-adjusted CSPAH rates per 100,000 population were 475.3 for NYC, 433.8 for non-NYC urban areas, 421.4 for suburban areas, and 332.0 for rural areas. (Table 2) In each urbanization group, the mean annual age-adjusted CSPAH rate increased as age increased, and those aged ≥80 years had >30 times the rate of CSPAH than those aged 20–44 years. (Table 2) The mean annual age-adjusted rate generally increased as area-based poverty level increased.

**Table 2:** Mean annual age-adjusted community-setting pneumonia-associated hospitalization rates in New York City, non-New York City urban areas, suburban areas, and rural areas—New York State, 2010–2014^a^.

	NYC		Non-NYC urban areas		Suburban areas		Rural areas	
Characteristic	Rate (95% CI)	RR (95% CI)	Rate (95% CI)	RR (95% CI)	Rate (95% CI)	RR (95% CI)	Rate (95% CI)	RR (95% CI)
Overall	475.3 (469.9, 480.6)	-- (--)	433.8 (429.4, 438.2)	-- (--)	421.4 (409.1, 434.0)	-- (--)	332.0 (319.1, 345.4)	-- (--)
Sex								
Female	417.5 (411.0, 424.1)	-- (--)	396.3 (390.6, 402.0)	-- (--)	383.6 (367.5, 400.3)	-- (--)	300.3 (283.4, 318.1)	-- (--)
Male	557.7 (548.7, 566.9)	1.3 (1.0, 1.6)	487.6 (480.5, 494.7)	1.2 (1.0, 1.5)	474.3 (454.8, 494.5)	1.2 (1.0, 1.5)	372.5 (352.4, 393.6)	1.2 (0.9, 1.6)
Age (years)^b^								
20–44	90.4 (87.2, 93.6)	-- (--)	86.0 (82.8, 89.3)	-- (--)	82.1 (72.9, 91.3)	-- (--)	64.5 (54.4, 74.6)	-- (--)
45–64	392.3 (383.9, 400.7)	4.5 (3.2, 6.3)	317.5 (311.0, 324.0)	4.5 (3.4, 6.0)	314.9 (296.4, 333.4)	4.3 (3.1, 6.0)	250.7 (231.1, 270.4)	3.9 (2.6, 5.8)
65–79	1,130.5 (1,106.9, 1,154.1)	13.1 (9.3, 18.4)	1,097.1 (1,077.2, 1,117.0)	12.9 (9.7, 17.1)	1,121.2 (1,065.3, 1,177.1)	14.2 (10.2, 19.7)	826.0 (770.8, 881.2)	13.4 (9.0, 19.9)
≥80	3,407.7 (3,342.0, 3,473.4)	38.9 (27.7, 54.6)	3,160.4 (3,108.9, 3,212.0)	33.1 (25.0, 43.8)	2,887.9 (2,744.0, 3,031.8)	35.8 (25.7, 49.8)	2,420.2 (2,263.7, 2,576.8)	41.0 (27.5, 61.2)
Area-based poverty level								
Low (<10% FPL)	317.8 (308.7, 327.0)	-- (--)	385.9 (380.6, 391.2)	-- (--)	275.5 (255.3, 297.0)	-- (--)	200.7 (182.6, 220.6)	-- (--)
Medium (10%–19% FPL)	445.7 (437.2, 454.3)	1.4 (1.2, 1.7)	463.7 (454.6, 473.0)	1.2 (1.0, 1.4)	441.2 (424.7, 458.2)	1.6 (1.4, 1.8)	374.6 (357.1, 393.0)	1.9 (1.7, 2.2)
High (20%–29% FPL)	553.5 (541.7, 565.6)	1.8 (1.5, 2.2)	556.8 (540.2, 573.8)	1.5 (1.3, 1.8)	558.9 (523.2, 596.7)	2.0 (1.7, 2.3)	474.1 (423.1, 530.2)	2.4 (2.0, 2.8)
Very high (≥30% FPL)	624.7 (609.8, 640.0)	2.2 (1.8, 2.7)	696.5 (669.8, 724.0)	1.9 (1.6, 2.2)	522.5 (432.5, 626.9)	1.9 (1.5, 2.4)	534.5 (340.2, 817.1)	2.5 (1.7, 3.7)

Abbreviations: CI, confidence interval; FPL, federal poverty level; NYC, New York City; RR, rate ratio.

^a^Rates are expressed per 100,000 population.

^b^Age group rates are not age-adjusted.

### CSPAH rates by urbanization level

After controlling for sex, age, and area-based poverty level, residents of non-NYC urban areas had 1.4 (95% CI, 1.3–1.6), residents of NYC had 1.3 (95% CI, 1.2–1.4), and suburban residents had 1.2 (95%, 1.1–1.3) times the rate of CSPAH compared with residents of rural areas (Table 3). McFadden’s pseudo R^2^ was 0.32.

**Table 3:** Multivariable negative binomial regression estimating community-setting pneumonia-associated hospitalization rate ratios—New York State, 2010–2014^a^.

Characteristic	Beta^b^	(SE)	RR	(95% CI)
Intercept	-7.807	(0.057)	--	--
Sex^c^				
Male	0.209	(0.029)	1.2	(1.2, 1.3)
Age (years)^d^				
45–64	1.434	(0.044)	4.2	(3.8, 4.6)
65–79	2.584	(0.044)	13.3	(12.2, 14.4)
≥80	3.632	(0.044)	37.8	(34.7, 41.2)
Area-based poverty^e^				
Medium (10%–19% FPL)	0.379	(0.039)	1.5	(1.4, 1.6)
High (20%–29% FPL)	0.599	(0.041)	1.8	(1.7, 2.0)
Very high (≥30% FPL)	0.747	(0.045)	2.1	(1.9, 2.3)
Urbanization group^f^^,^^g^				
NYC	0.252	(0.047)	1.3	(1.2, 1.4)
Non-NYC urban areas	0.356	(0.047)	1.4	(1.3, 1.6)
Suburban areas	0.200	(0.050)	1.2	(1.1, 1.3)

Abbreviations: CI, confidence interval; FPL, federal poverty level; NYC, New York City; RR, rate ratio; SE, standard error.

^a^McFadden’s pseudo R^2^ was 0.32.

^b^The p-values are all <0.01.

^c^The reference group is female.

^d^The reference group is age 20–44.

^e^The reference group is low area-based poverty (<10% FPL).

^f^The reference group is rural areas.

^g^Urbanization groups across New York State were classified using Rural-Urban Continuum Codes (RUCC) based on county of patient residence in NYC (RUCC 1), non-NYC urban areas (RUCC 1–3 other than NYC), suburban areas (RUCC 4–5), or rural areas (RUCC 6–8).

## Discussion

Given that published data on pneumonia rates in different parts of NYS are limited, despite the reported higher pneumonia death rate in NYC compared with the rest of NYS, our objective was to compare CSPAH rates between urbanization groups across NYS [1, 2]. We found that NYC had a higher mean annual age-adjusted CSPAH rate than other parts of NYS, as well as the highest proportion of CSPAH with in-hospital death. Non-NYC urban areas had the second highest CSPAH rate, followed by suburban and finally rural areas. After controlling for age, sex, and area-based poverty level, the CSPAH rate was higher for all urban (both NYC and non-NYC) and suburban areas versus rural areas.

It is unlikely that the higher CSPAH rates in NYC and non-NYC urban areas were driven by HIV status or COPD, both of which are associated with pneumonia. Because only 2.8% of CSPAH in all urban areas of the state, including NYC, were associated with HIV/AIDS, lower hospital admission thresholds for patients with HIV infection were unlikely to have a major effect on the increased CSPAH rate [26]. HIV will hopefully be an even lower driver of CSPAH rates in NYS in the future given the statewide Ending the Epidemic initiative [27]. The proportion of CSPAH patients with COPD, which is strongly correlated with smoking, was lower in urban areas of NYS compared with non-urban areas, and therefore COPD is likely not driving the higher CSPAH rate in the former [28]. The low prevalence of COPD among CSPAH patients in NYC is expected given the lower prevalence of current smokers in NYC versus the rest of the state, and this likely reflects NYC’s history of aggressive tobacco control [29]. While COPD did not drive the higher CSPAH rates in urban areas, COPD prevalence was high in all urbanization groups of NYS including NYC (approximately 1 in 5 CSPAH in NYC were associated with COPD). Efforts to promote smoking cessation remain important because they may decrease COPD and thus risk of CSPAH across the board, even if they may not affect the differential rates of CSPAH.

Conversely, we found a greater burden of CSPAH among residents of higher-poverty-level areas in NYC compared with the rest of the state. Most strikingly, 1 in 5 CSPAH in NYC occurred in residents of high-poverty-level neighborhoods versus approximately 1 in 100 in rural areas. In addition, the proportion of deaths during CSPAH experienced by patients residing in high- and very-high-poverty-level areas was greatest in NYC. Previous studies have established a relationship between poverty and pneumonia hospitalization and mortality [6, 20, 30]. This relationship may be partly due to higher prevalence of chronic diseases such as asthma, COPD, and diabetes in impoverished areas that increase pneumonia risk [31–34]. The NYC crowding (i.e., housing unit occupied by ≥1 person per room) rate in 2013 of 8.8% was more than double the national rate, which may have played a role in increasing transmission of common pneumonia-causing agents [35]. The bottom income quartile in NYC is disproportionately impacted by crowding compared with the top quartile (23.6% versus 18.5%, respectively), and overcrowded living situations could contribute to the association between poverty and risk of hospital admission in combination with other factors, including limited financial ability to seek or afford healthcare while in the earlier stages of illness [35, 36]. Improvements in incidence and outcomes of community-setting pneumonia may be obtained by ensuring that impoverished communities have access to appropriate health education and preventive care.

A potential reason why a higher proportion of CSPAH led to in-hospital death in NYC than elsewhere in the state is that a higher proportion of patients in NYC may have had more severe or difficult-to-treat pneumonia. While we cannot confirm this without further validation such as reviewing medical charts or calculating the Pneumonia Severity Index, almost 10% of CSPAH in NYC lasted ≥21 days compared with <6% in other areas of NYS. Longer hospital stays would suggest worse health status possibly caused by more severe pneumonia. Of note, we calculated van Walraven comorbidity index scores for risk of in-hospital mortality and found similar results across all urbanization groups, but these scores reflect the burden of critical comorbid conditions. They do not specifically reflect pneumonia severity [37]. Another possible explanation for the greater burden of deaths during CSPAH in NYC is related to antimicrobial resistance, which can lead to more difficult-to-treat pneumonias. NYC had a higher proportion of CSPAH and deaths during these hospitalizations that were HCAP- versus CAP-related than suburban and rural areas of the state, and certain HCAP criteria, such as admission from a nursing home, may be more strongly associated with antimicrobial resistance than CAP [38].

### Limitations

Although our study was able to access a rich source of NYS hospitalization data, it has some limitations. First, SPARCS is restricted in the type of information it provides on inpatient stays. We were unable to determine whether hospitals differed from one another in their threshold for admitting pneumonia patients, severity of pneumonia in admitted patients, and methods of pneumonia diagnosis. We were also unable to obtain microbiologic data to investigate whether some areas had a greater burden of pneumonia due to antimicrobial-resistant pathogens. Second, we did not verify the accuracy of SPARCS data. Accuracy of SPARCS race/ethnicity data may especially be limited, and caution should be taken when drawing inferences from the race/ethnicity data presented here [39, 40]. Third, differences across urbanization groups in CSPAH rates as well as in prevalence of comorbidities among patients may have been influenced by coding bias specific to certain areas of NYS. Fourth, our study focused on hospitalized patients, and therefore we cannot draw conclusions for CAP and HCAP in the outpatient setting. Fifth, we were unable to adjust for important confounders such as smoking history, household income (versus area-based poverty), education level, and neighborhood pollution in our final model because stratified population denominators were unavailable. The low RRs could be a result of residual confounding. Lastly, while the data presented here are several years old, we chose to only include data through 2014 because the ICD-9-CM system was phased out in 2015. Unfortunately, since 2014, pneumonia continues to be a leading cause of death across NYS [1, 2].

## Conclusions

We used administrative discharge data to estimate mean annual age-adjusted CSPAH rates and proportion of CSPAH with in-hospital death in NYS residents aged ≥20 years by urbanization level during 2010–2014. While our study does not explain why a greater proportion of CSPAH resulted in death in NYC versus other urbanization groups, it suggests that poorer urban communities and the elderly may especially benefit from strategies to reduce CAP and HCAP. Future investigations would ideally have access to data with more robust race/ethnicity data than is available in SPARCS and examine CSPAH rates by dual minority status (e.g., Black urban residents). These populations can be more vulnerable to adverse effects of infectious respiratory diseases due to longstanding structural barriers to achieving optimal health outcomes, as has been demonstrated by the current coronavirus outbreak, during which NYC emerged as an epicenter and Black and Hispanic residents both across the state and in NYC suffered from disproportionate fatality rates [41, 42]. Additional clinical data (e.g., microbiologic data) and information about capacity of care in different areas of NYS (e.g., number of facilities, number of beds) would be valuable in better understanding the epidemiology of pneumonia, especially as related to in-hospital death.
